# Teaching how to cite scientific articles: a study of citation deviation in citing "multi-authored papers" by top authors from Iranian Universities of Medical Sciences in 2017 

**Published:** 2020

**Authors:** Zahra Hosseini Ahangari, Abedin Hosseini Ahangari, Mohamad Alaae Arani

**Affiliations:** 1 *Omid Counseling Center, Amol, Iran*; 2 *Medical Library & Information Science, Abadan School of Medical Sciences, Abadan, Iran *; 3 *University of Kashan*

**Keywords:** Citation behavior, Self-Control, Moral development, Multi-authorship

## Abstract

**Aim:**

The present study aims at teaching the right citation models in scientific publications by top authors from Iranian universities of medical sciences in 2017, considering the relationship between moral development and self-control variables and model of "citation to multi-authored papers" in these articles.

**Background:**

Multi-authorship increases the amount of self-citation and also increases the likelihood of being cited by others.

**Methods:**

This study is of the applied scientometrics and correlation (model presentation) studies type. The research population in the first part of this study includes all the authors who had an H-index of 10 or more in the scientific databases of 2017. The sample size at this stage was 110, selected by systematic random sampling. The collected data were analyzed using SPSS 16.0.

**Results:**

The results of regression analysis based on the concurrent method indicated that the regression model is significant. The coefficient of determination is 0.096 and the F ratio is 5.650, which are significant at the level of p <0.001. In this regression model, the variables of the level of moral development (β = -5.801, p <0.001) and self-control (p < 0.001, β = -0.253) have significant predictive power and can be considered as predictors of behavioral modeling in citing "multi-authored papers".

**Conclusion:**

Based on the results, teaching how to avoid blindly citing the "multi-authored papers", which is regarded as a kind of "citation deviation", can, to some extent, lead to strengthening citation indexes.

## Introduction

 The author's prominence, the journal's prominence (1-4), the prominence of the article in terms of the citations made (5), and the number of contributing authors (Beaver, 2004) are the social causes that shape authors' citation motives.

Researchers have different views on these conflicting motivations. Merton views the scientist primarily as a seeker of scientific truth without any expectations and believes that citation reflects the cognitive or logical effects of a scientific work (6), while Cronin claims that citation is not a phenomenon that occurs in a vacuum and is not separate from its productive context and background. (7).

Cronin rejects Merton's view that citation is dominated by a number of universally recognized and specific norms, but at the same time argues that the process of citation is not coincidental. He distinguishes between the "internalized" and "externalized" approaches to citation analysis. The first approach looks at the quantities and the frequency distributions, while the second one focuses on the contexts and processes in light of which authors collect their cited references. A combination of these two approaches is needed to gain a deeper understanding of what citations measure.

Gilbert (1977), a British sociologist and advocate of the social structure theory of citation behavior (Cronin's theory), believes that citation is a means of persuading the audience (8). Gilbert describes it as follows; a scientist who has come up with results that he trusts seeks to persuade the scientific community about it and provides evidence that is believed to be credible so that the audience is thus persuaded. (9).

Bornmann and Daniel (2008) also argue that the motivations for citing in scientific societies are not just based on the cognitive and logical effects of scientific peers, but are in some cases purely non-scientific or for social reasons (10). In view of the foregoing, the question arises whether, from a psychological point of view, other factors will lead to different motivations in the author's citation process. In other words, how are authors' citation motives explained? Cronin is one of the theorists who emphasizes the potential of the psychological approach to the study of citation, in particular, examining the relationship between cognitive style and personality. In this regard, this study also attempts to examine non-scientific or social reasons and motivations within the framework of moral development level and self-control as part of individuals' personality traits.

The purpose of this research is to study the level of moral development and self-control as social and psychological variables affecting the citation motives in the model of "citation to multi-authored papers" by top Iranian authors in the field of medical sciences in 2017. The term "multi-authored papers" denotes articles written by more than six authors at the time of publication. 

Objectives

• Assessing the status of the model of "citation to multi-authored papers" by Iranian medical sciences top authors in 2017

• Assessing the level of moral development and self-control of Iranian medical sciences top authors

• Assessing the impact of the level of moral development and self-control on the model of "citation to multi-authored papers" by Iranian medical sciences top authors

• Providing solutions and practices for modifying the citation behavior of Iranian medical science top authors as citation behavior models are a type of educable behavior and can be modified by the individual's cognitive development during training. Thus, the purpose of this study is to investigate the behavioral model of "citation to multi-authored papers" by Iranian medical sciences top authors. This can be helpful in formulating educational and research policies in the Ministry of Health and Medical Education in Iran.

• Determining the severity and type of relationship between the level of moral development and self-control on the model of "citation to multi-authored papers" by Iranian medical sciences top authors can provide a proposed model of the relationships between the abovementioned variables in explaining the citation behavior towards "multi-authored papers" and appropriate solutions to prevent deviation.

## Methods

In the present study the researchers seek to examine the relationship between the scientology and social psychology variables of a basic-applied type (model presentation). In order to test the research hypothesis, the study was conducted in two separate sections. In the first part of the study, DIT questionnaires and the Tanji Self-Control Questionnaire (Questionnaire-short Form) were employed to measure the level of moral development and self-control of the authors, respectively. The second part was done by the citation analysis method, so that two articles from each sample member would be studied by the citation analysis method. The population in the first part of this study included all authors who had an H-index of 10 and more in the 2017 Scopus database. In addition to the author's responsibility, as a selection criterion, only the authors' most recent publications (the researchers’ two recent articles) were chosen. Sampling was conducted by systematic random sampling method using Krejcie and Morgan table. The sample size at this stage was 110 subjects according to n = 104 + m formula and the study background. The sample size was selected based on the statistical method of research, i.e., regression and correlation.

Similar research has been done with smaller samples. For example, Donald Case and Joseph Miller (2011), in their study on the citation motivations of bibliographic authors, benchmarked 6 highly cited papers and studied the authors who cited these articles in terms of citation motivation (11). The number of authors was 102. The data collection tool was DIT questionnaires and the Tanji Self-Control Questionnaire (short form) in 13 components. The sample size at this stage was 220 articles (Two articles per author). The selection of two articles for each individual was based on the background of the citation behavior research. In past studies, in order to investigate the author's citation motives and behaviors, one or two articles of the criterion authors were usually examined. Vinkler (5) examined 20 papers in his research on the citation motivations of 20 authors in the field of chemistry (each author had one article and a total of 484 citations). After the purposeful selection of 220 articles from the articles of the study subjects, all the references of the desired articles were analyzed in terms of citation for extraction of variables in the second part of the study. The articles reviewed had a total of 8882 citations (an average of 40.4 citations per article). The data collected during the first and second parts of the study were analyzed using SPSS 16.0. Pearson correlation and multiple regression were used to analyze and test the research hypotheses. Regression analyses were performed based on the enter method.

**Research** **Questions**

1. Is there a relationship between the level of moral development and self-control and the model of "citation to multi-authored papers" by top authors from Iranian universities of medical sciences?

2. To what extent is the model of "citation to multi-authored papers" affected by the level of moral development and self-control variables?

**Hypothesis**

1. The level of moral development and self-control variables can predict the model of "citation to multi-authored papers" by top authors from Iran universities of medical sciences. 

## Results

**Figure 1:**
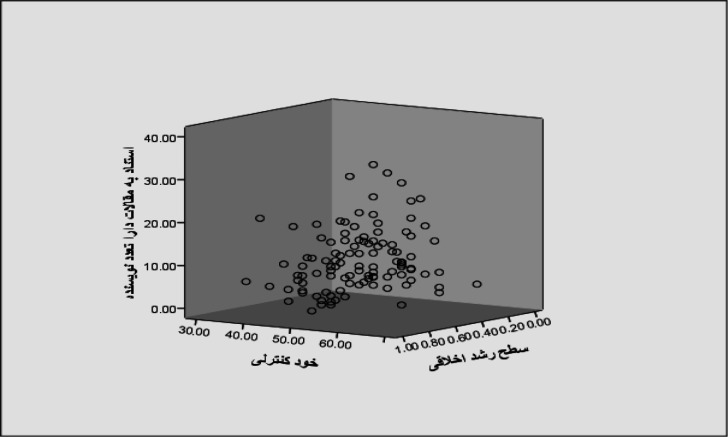
The communication model in the model of "citation to multi-authored papers

**Figure 2:**
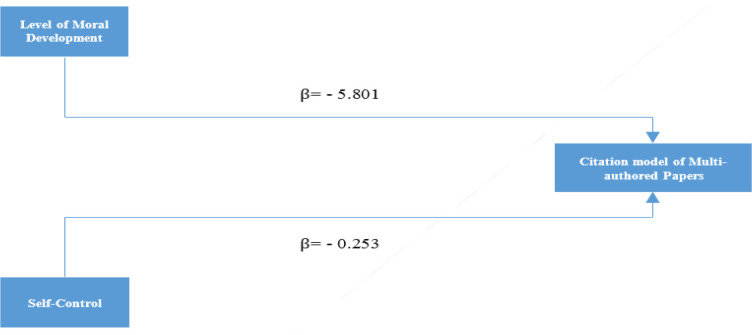
Predicting the model of Citation to Multi-authored Papers as Affected by Variables of Moral Development Level and Self-Control

The results of the regression analysis based on the enter method indicate that the regression model is significant. As can be seen in Table 1, the coefficient of determination is 0.096 and the F ratio is 5.650, which are significant at the level of p <0.001. Table 1 figures 1and 2 show that in this regression model, the variables of the level of moral development (β = -5.801, p <0.001), and self-control (p < 0.001, β = -0.253) have significant predictive power and can be considered as a predictor of behavioral model in citing "multi-authored papers". According to the regression coefficients, the variable of the level of moral development has a greater effect on the citation model of multi- authored papers than the self-control variable. The coefficient of determination in this relation is 0.096 and indicates that 9.6% of the variations in the model variable of "citation to multi-authored papers" can be predicted by the variables predicting the level of moral development and self-control (Table 1). Regression equation: the way to predict the variable of citation to multi-authored papers based on two variables predicting the level of moral development and self-control can be shown in the regression equation:

Y=∝+(β_1_X_1_) + (β_2_X_2_)

*Y*= 26/739 – 5/801 X_1_ – 0/253 X_2_

In this equation, the variables of the level of moral development and self-control simultaneously have significant predictive power. As can be seen, there are two predictor variables and one criterion variable in this equation. The two predictor variables x1 and x2 are two factors of the level of moral development and self-control, respectively. The value ∝ or y-intercept is 26.739 based on regression analysis. The coefficients of β1 and β2 are -5.801 and -0.253, respectively. Negative values ​​indicate the inverse relationship between the predictor and criterion variables. We can thus calculate the variable values ​​of citation to multi-authored papers based on the values ​​of two variables of the level of moral development and self-control. The higher factor coefficient of the moral development indicates this variable is more important in predicting the criterion variable.

## Discussion

Regarding the model of "citation to multi-authored papers", it was observed that there were, on average, 10.75 articles with more than 6 authors cited from, in each article by the authors studied in the present article. The lowest number of citations from the “multi-authored papers" was 0 and the highest number was 30.50.

Glänzel and Thijs (2004) have shown that multi-authorship increases the amount of self-citation (and increases the likelihood of being cited by others as well) (12). Costas et al. (2010) and Aksnes (2003) also report that the number of self-citations increases the number of author citation sources (13, 14). An increase in the authors' number of citations can lead to more citations in the document of the author. According to the findings of this study regarding the average number of citations from multi-authored papers in the studied articles, it should be noted that considering 10.75 citations per article based on the average number of "total citations" per article – i.e. about 40 citations per article – it can be concluded that 25% of the received citations in articles by top authors from Iranian universities of medical sciences are from articles with more than 6 authors, indicating that they have accepted the unscientific assumption that the larger the number of authors, the better the quality of the article; or perhaps in recent years authors prefer to work in a more collaborative environment. Authors might show a strong inclination towards writing interdisciplinary articles where authors from different fields of science could collaborate. In addition, the findings of this study indicate that there is a significant inverse linear correlation between the self-control variable and the model of "citation to multi-authored papers", which means that with an increase in self-control level, the rate of citation to “multi-authored papers” decreases. Confirming the findings of the present study, 

**Table 1:** Results of Multiple Regression between the variables of the level of moral development and self-control and the model of "citation to multi-authored papers" by the Enter Method

Criterion Variable	Regression Coefficients	F Ratio (P) Probability	Coefficient of Determination RS	Statistical Indicators Predictor Variable		
				1	2	
Model of "Citation to Multi-Authored Papers"	Level of Moral Development	F= 5.650 P= 0.005	0.096	β= - 5.801 t= - 2.593 P= 0.011	β= - 0.253 t= - 2.190 P= 0.031	2.089
	Self-Control					

Beaver (2004) found that there is a positive correlation between the number of citations made to an article and the number of contributing authors in the compilation of an article (15). That is, the greater the number of authors of an article, the more citations it will receive in the future, and it is the social and psychological pressure on the author that reinforces this assumption in the author's mind that: written articles by several authors are certainly of a higher quality. Based on these interpretations, the findings of this study suggest that there is a relationship between the level of moral development and self-control and authors' citation behavior models and the predictive power of these behavior models through the abovementioned factors, confirming the social structure theory of citation behavior. In other words, some of the author's citation behaviors are influenced by social pressures, and the context in which the citation process occurs is influential in this process. These findings also explicitly contradict the normative theory of citation behavior, which claims that citation merely reflects the cognitive or logical effects of a scientific work.

**Predicting the Model of "Citation to Multi-authored** **Papers** **" in** **the** **Citation Behaviors of** **Iranian** **Top Authors** **in the Field of Medical Sciences** **Using the level of Moral Development and Self-Control**

The analytical and final findings of the current study show that the regression analysis based on the enter method indicates that the regression model is significant.

In this regression model, both variables of the level of moral development (β = -5.801, p <0.001, and self-control (β = -0.253, p <0.001) have significant predictive power and can be considered as a predictor of the model of “citation to multi-authored papers”. According to the regression coefficients, the variable of the moral development level has a greater influence on the model of "citing multi-authored papers" than the self-control variable. The coefficient of determination in this regard is 0.096 and indicates that about 10% of the variations in the model of "citation to multi-authored papers" can be predicted by the variables predicting the level of moral development and self-control. In other words, 10% of the level of moral development and self-control are the main reasons for the tendency of authors to refer to multi-authored papers. Based on this finding, the hypothesis is confirmed.

According to the coefficients obtained by assuming that the effect of the self-control variable is constant for a unit increase in the level of moral development, the average unit of "citation to multi-authored papers" variable is reduced to -5.801 and assuming that the effect of moral development level variable is constant, per unit in the self-control variable increases to -0.253, the variable mean unit of " citation to multi-authored papers" decreases.

The multiplicity of authors is one of the topics that has received much attention in scientometrics and scientific interactions. It seems that multi-authored papers have a higher validity and this has affected the policies adopted by journals to publish articles, as multi-authored articles have a higher acceptance rate. The findings of Smart and Bayer's (1986) study indicate that multi-authored papers have higher acceptance rates than individual papers (16). Smart and Bayer argue that there is a link between multi-authorship and the quality of an article and multi-authored articles are thus more widely cited. Beaver (15) also indicated in a study that there was a positive correlation between the number of citations made in an article and the number of contributing authors in an article. It can be concluded that multi-authored articles are often of higher quality and as a result authors refer to such articles more often. This has also led to an increase in the tendency of authors to write multi-authored papers in recent years. One of the important reasons leading to authors citing multi-authored papers which consequently constitutes a moral development level and self-control is the desire to be persuasive (17, 18). Nigel Gilbert (8), a British sociologist and a supporter of the social structure theory of citation behavior, believes that citation is a means of persuading the audience. Gilbert holds that a scientist who has come to a conclusion that he believes in tries to persuade the scientific community to accept it and provides evidence in this regard that is believed to be authentic so that the audience would be persuaded (9). In other words, the authors choose behaviors in their citation that can better satisfy the audience. Citing multi-authored papers in the context of moral development level and self-control is also explained in this context.

## Conflict of interests

The authors declare that they have no conflict of interest.

## References

[1] Shadish, WR, Tolliver D, Gray M, Sengupta SK (1995). Author judgments about works they cite-three studies from psychology journals. Soc Stud Sci.

[2] Balaii H, Asadzadeh Aghdaei H, Farnood A (2015). Time trend analysis and demographic features of inflammatory bowel disease in Tehran. Gastroenterol Hepatol Bed Bench.

[3] Walters GD (2006). Predicting subsequent citations to articles published to twelve crime psychology journals. Scientometrics.

[4] Haslam N, Ban L, Kaufmann L, Loughnan S, Peters K, Whelan J (2008). What makes an article influential? Predicting impact in social and personality psychology. Scientometrics.

[5] Vinkler P (1987). A quasi-quantitative citation model. Scientometrics.

[6] Merton RK (1988). The Matthew effect in science, II: cumulative advantage and symbolism of intellectual property. ISIS.

[7] Cronin B (1984). The Citation process: The role and significance of citations in scientific communication.

[8] Gilbert GN (1977). Referencing as persuasion. Soc Stud Sci.

[9] Glänzel W, Thijs B (2004). Does co-authorship inflate the share of self-citations?. Scientometrics.

[10] Moed HF, Garfield E (2004). In basic science the percentage of authoritative references decreases as bibliographies become shorter. Scientometrics.

[11] Bornmann L, Daniel HD (2008). What do citation counts measure? A review of studies on citing behavior. J Doc.

[12] Javanmard E, Niyyati M, Ghasemi E, Mirjalali H, Asadzadeh Aghdaei H, Zali MR (2018). Impacts of human development index and climate conditions on prevalence of Blastocystis: A systematic review and meta-analysis. Acta Trop..

[13] Costas R, Van Leeuwen TN, Bordons M (2010). Self-citations at the mesa and individual levels: Effects of different calculation methods. Scientometrics.

[14] (2003). Aksnes D. A macro study of self-citation. Scientometrics.

[15] Beaver DB (2004). Does collaborative research have greater epistemic authority?. Scientometrics.

[16] Smart JC, Bayer AE (1986). Author collaboration and impact: Citation rates of single and multiple authored articles. Scientometrics.

[17] Geravandia S, Sahebalzamani M, Adhami Moghadam F, Mehrpour M, Yousefi F, Hoseini Ahangari SA (2019). Refusing to report the medication errors observed in Ahvaz Jundishapur University of Medical Sciences during 2014–2015. Clin Epidemiol Global Health.

[18] Soltani F, Geravandi S, Mohammadi MJ, Alizadeh R, Valipour A, Hoseini A (2018). The Effect of Education on the Nursing Care Quality of Patients who are under Mechanical Ventilation in ICU ward. Data in Brief.

